# Antigens and Antibodies of the Antiphospholipid Syndrome as New Allies in the Pathogenesis of COVID-19 Coagulopathy

**DOI:** 10.3390/ijms23094946

**Published:** 2022-04-29

**Authors:** Manuel Serrano, Gerard Espinosa, Antonio Serrano, Ricard Cervera

**Affiliations:** 1 Department of Immunology, Healthcare Research Institute I+12, Hospital 12 de Octubre, 28041 Madrid, Spain; mserranobl@gmail.com; 2 Department of Autoimmune Diseases, Hospital Clínic, Insititut d’Investigacions Biomèdiques August Pi i Sunyer (IDIBAPS), Universitat de Barcelona, 08036 Barcelona, Spain; gespino@clinic.cat (G.E.); rcervera@clinic.cat (R.C.)

**Keywords:** antiphospholipid syndrome, COVID-19 coagulopathy, antiphospholipid antibodies, B2GP1 deficiency

## Abstract

High prevalence of both criteria and extra-criteria antiphospholipid antibodies (aPL) has been reported in COVID-19 patients. However, the differences in aPL prevalence decreased when an age-matched control group was included. The association of aPL with thrombotic events in COVID-19 is very heterogeneous. This could be influenced by the fact that most of the studies carried out were conducted on small populations enriched with elderly patients in which aPL was measured only at a single point and they were performed with non-standardized assays. The few studies that confirmed aPL in a second measurement showed that aPL levels hardly changed, with the exception of the lupus anticoagulant that commonly reduced. COVID-19 coagulopathy is an aPL-independent phenomenon closely associated with the onset of the disease. Thrombosis occurs later in patients with aPL presence, which is likely an additional prothrombotic factor. B2-glycoprotein deficiency (mainly aPL antigen caused both by low production and consumption) is very common during the SARS-CoV2 infection and has been associated with a greater predisposition to COVID-19 complications. This could be a new prothrombotic mechanism that may be caused by the blockage of its physiological functions, the anticoagulant state being the most important.

## 1. Introduction

COVID-19 is a disease caused by SARS-CoV2 infection whose course is heterogeneous and unpredictable [1]. Most patients suffer from the mildest form, with flu-like symptoms that are often so mild that the disease can go unnoticed [2]. Around 15% of the patients infected develop severe manifestations, including unilateral or bilateral pneumonia with acute respiratory distress syndrome (ARDS) and progressive hypoxemia that may require mechanical ventilation assistance. Systemic hyperinflammation occurs in its severest form, with multiorgan involvement (cytokine storm), lymphopenia, and marked elevation of C-reactive protein, ferritin, D-dimers, cytokines and chemokines [3,4], which can be life threatening.

Although COVID-19 is principally a respiratory disease, it also acts on the cardiovascular level and causes thrombotic events mainly in the arteries/arterioles, microcirculation and venous system [5,6]. These events appear more frequently in acute infection, but they can also occur during convalescence [7]. Autopsies in COVID-19 deceased patients have shown microthrombi, diffuse alveolar damage, multiorgan thrombosis, hemophagocytosis and immune cells depletion [8]. Coagulation disorder is relatively common in COVID-19 patients and can be present in approximately 50% of those patients whose stay in the Intensive Care Units (ICU) is two weeks or longer. Most of the thromboses that appeared were located in the lungs and were independent of whether the patients had received standard-dose thromboprophylaxis (87%) [9].

The parameters related to thrombogenesis, such as D-dimer, fibrin, C-reactive protein levels, lactate dehydrogenase (LDH) and moderate thrombocytopenia, are usually elevated in patients affected by COVID-19 coagulopathy. Therefore, the infection would constitute an additional contributing factor that would lead to a highly prothrombotic state.

Our study methodology was based on the answer to the question “Are the antigens and antibodies of the antiphospholipid syndrome two more allies in the COVID-19 coagulopathy”. We made a search for the published evidence on the antigens and antibodies of the antiphospholipid syndrome related to the pathogenesis of COVID-19 coagulopathy. The key words used for the search were, among others, antiphospholipid syndrome; COVID-19 coagulopathy, and Antiphospholipid antibodies. The prevalence and types of aPL (including extra-criteria aPL), confirmation after 12 weeks and the association with thrombosis were analyzed.

## 2. Immunothrombosis

Coagulopathy in coronavirus infections may be caused not only by several direct mechanisms such as endotheliitis with elevated levels of von Willebrand factor, systemic inflammation, due to activation of the Toll-like receptor, and activation of the tissue factor pathway [10], but also by indirect mechanisms such as: acute respiratory distress syndrome (ARDS) and endotheliosis tissue hypoxia caused by incorrect diffusion of gases [11]; hypoxia caused by ARDS activates several transcriptional changes in cells that proceed to elaborate hypoxia-inducible transcription factors (HIF-1 and HIF-2) which, in turn, increases thrombin levels [12]; the large number of apoptotic cells that are generated as a consequence of infection or sepsis [13] increases proinflammatory responses that can cause ARDS and thrombosis [14]; and the strong inflammatory environment secondary to COVID-19 infection induces expression of coagulant factors and integrins that implies activation of platelets and immune cells like neutrophils triggering coagulopathy and thrombi formation (immunothrombosis) through the neutrophils extracellular traps (NETs) pathway [15].

NETs are three-dimensional extracellular networks of decondensed chromatin, histones and antimicrobial proteins with the ability to trap and kill microbes as part of the innate immune system, thus preventing their spread and concentrating the antimicrobial factors at the site of infection [16]. The histones and enzymes released from NETs have cytotoxic activity that produces endothelial dysfunction and cell death called NETosis mediated by neutrophils [17]. In this way, the release of NETs acts as an inflammatory amplifier enabling self-antigen exposure and autoantibody production [18]. This activity reinforces the relevance of NETs in pathological processes other than thrombosis, such as chronic aberrant immunity and long-term COVID-19 [19].

NETs, which promote thrombus formation, have already been described in settings such as deep vein thrombosis, stroke and myocardial infarction [20]. In relation to COVID-19, NETs have been shown to contribute to the formation of microthrombi through platelet-neutrophil interactions in patients with respiratory distress [21].

Moreover, the complement system usually plays an important role in the context of inflammation, thrombosis and activation of the innate response. Complement deposits have been reported in the lung and skin tissue, which suggests systemic activation of both the classical and lectin-based complement pathways [22,23]. These findings suggest a multiorgan vascular disease overlapping with a thrombotic microangiopathy, such as TMA or PNH, in which complement overactivation plays an important role in the pathophysiology of thrombosis [24,25].

## 3. APS and Thrombosis

The COVID-19 prothrombotic environment within the context of a strong inflammatory response is reminiscent of antiphospholipid syndrome (APS), especially in its catastrophic form [26,27]. APS is a systemic autoimmune disease characterized by thrombosis and/or pregnancy morbidity associated with the presence of antiphospholipid antibodies (aPL).

Nowadays, there are no defined diagnostic criteria for APS, although there are widely accepted classification criteria that have been used in some situations for its diagnosis despite their low sensitivity. To classify a patient as thrombotic APS, the concurrence of a clinical criterion (thrombosis) and a laboratory criterion (positivity of one or more aPL) is required [28]. The aPL included in the classification criteria are lupus anticoagulant (LA), anticardiolipin (aCL) and anti-β-2-glycoprotein I (aB2GP1) antibodies of IgG and/or IgM isotypes.

aPL levels can rise temporarily and nonspecifically during acute infectious episodes [29]. A second measurement, at least 12 weeks from the first, is needed to avoid possible false positives [28].

There are three main presentations of APS: (1) that associated with another autoimmune disease (such as systemic lupus erythematosus), (2) primary APS without any other associated disease, and (3) catastrophic APS (CAPS), a life-threatening variant characterized by the development of multiple thrombosis in a short period of time, poor response to anticoagulant treatment and a high risk of failure of function of several vital organs. The third presentation is a situation that is very similar to COVID-19 coagulopathy [30].

### Extra-Criteria APS Clinical Features and Autoantibodies

In addition to the clinical characteristics included in the classification criteria, there are others that have not been included in the clinical classification criteria despite being very common. These include livedo reticularis or thrombocytopenia [31]. The same occurs with other aPL that have not been included in the classification criteria. The most prevalent are the anti-phosphatidylserine/prothrombin antibodies (aPS/PT) and aB2GP1 antibodies of IgA isotype. aPS/PT antibodies have a strong correlation with thrombotic events and the presence of LA [32]. IgA aB2GP1 antibodies have also been associated with a higher risk of thrombosis, this being especially prevalent in patients with chronic kidney disease and end-stage heart failure. IgA aB2GP1 antibodies have been strongly associated with thrombosis, morbimortality and graft loss after heart and renal transplantation [33,34].

Other widely used extra-criteria aPL are the antibodies against domain I of B2GP1 (IgG) that have high specificity (97.12%) but moderate sensitivity (64.32%) for the diagnosis of APS [35,36].

Even though the causes that lead to the production of aPL are unknown, the influence of microbial and viral agents that suggests an infectious etiology has been observed [37]. This could be explained by molecular mimicry between the B2GP1 and some molecular structures of several microorganisms [38]. This mimicry occurs in some predisposed individuals whose self-tolerance mechanisms fail and produce an abnormal response because they are not capable of considering molecular structures similar to microbial peptides of their own [39]. Therefore, the steady state would not be restored after the resolution of the infection, and the presence of autoantibodies would be maintained.

The mechanism of thrombosis-induction by aPL is also not fully understood. The “two hits” theory is the most accepted model on how thrombosis occurs. In this model, the presence of aPL (first hit) is necessary but not enough to trigger a thrombotic event. Another factor (second hit) that implies an activation of innate immunity, such as inflammation, infection, or surgery, is required to trigger the thrombotic event [40].

## 4. aPL in COVID-19

During the first weeks of the pandemic, some reports associated the prothrombotic state of COVID-19 with the presence of aPL [41]. The first study reported the presence of thrombotic events in three patients with the presence of aPL of IgG and IgA isotypes. 

Based on a systematic screening for aPL in COVID-19 patients with no past history of APS, it was found that numerous studies reported an elevated prevalence of aPL (Table 1). 

*Criteria aPL*: The most prevalent was LA: 50–90% of COVID-19 patients were LA positive [42]. LA positivity was 91% among patients who had elevated aPTT [43]. 

The prevalence of aCL and aB2GP1 antibodies of IgG/M isotypes is around 15% which is lower than the LA prevalence. The simultaneous presence of several aPL, especially that of the double positivity that occurred in 25–50% of patients, is also frequent [44]. 

No significant differences have been observed regarding prevalence in the studies that determine aCL and aB2GP1 antibodies of the IgG/IgM isotypes in COVID-19 patients. This could be due to the fact that the diagnostic kits are very well standardized and there is a great deal of experience with these antibodies. Most published studies evaluated small patient samples, which could be considered a handicap when calculating statistically significant associations. 

*Extra-criteria aPL*: Although the data on prevalence are very heterogeneous, there is a strikingly high prevalence of data on aPL. Only a few single-center studies have performed complete screening for extra-criteria aPL (aPS/PT IgG and IgM, aB2GP1 and aCL of the IgA isotype). Zuo et al. reported high prevalence for aPS/PT of the IgG and IgM isotypes, with 24% and 18%, respectively [44] and IgA aB2GP1 and aCL were present in <5% of patients. On the contrary, Borghi et al. reported a prevalence of extra-criteria aPL under 10%. In addition, they reported that only 5% of patients with aPL recognize the aB2GP1 domains I or IV/V [27]. 

Only two multicenter studies have been found. One of them, that performed by Gatto et al., conducted aPL screening on a population of 122 patients, only 52 of whom were hospitalized and in whom 18 events were recorded. The most frequent aPL were aCL IgG and LA, however, the study did not find a statistical association or higher prevalence than in the population with primary APS or with other systemic autoimmune diseases [45]. However, the sample size of patients with aPL positive was too small to establish a valid statistical association. 

The other multicenter study found has a larger sample. It included 474 patients who had 35 thrombotic events during their follow-up. The prevalence for any aPL was 23.6%. IgA aB2GP1 antibodies were the most prevalent aPL with 15% positivity. In addition, they only found significant differences in prevalence with the aPL when they compared their sample with a reference population of similar age [46].

Other studies conducted on critically ill patients have shown that 19% had high prevalences of IgM anti-annexin antibodies [44]. Prevalences of up to 30% of IgA aPL, both aCL and aB2GP1, have also been reported [47] and their presence has been associated with the most severe cases of COVID-19 [48].

## 5. Clinical Implications of aPL in COVID-19

At present, the information regarding the pathogenicity of aPL during the SARS-CoV2 infection is heterogeneous. Some studies have described an association between aPL and disease severity and have found a higher prevalence in patients with ARDS, lower glomerular filtration rate and more ICU requirement [47,49,50]. Furthermore, the pathogenicity of IgG aPL has been demonstrated in an animal model [51]. Likewise, COVID-19 patients with multiple aPL positivity had a significantly higher incidence of ischemic stroke compared to patients who were negative (*p* = 0.023), the most prevalent being the aPL of IgA isotype [47]. In addition, a prospective study with 361 patients showed an association between the presence of aPL and incidence of thrombosis in the first six months after COVID-19 diagnosis OR: 3.7, 95% CI (1.7–8.1) [52]. In one multicenter study, IgG aB2GP1 was the only aPL that showed an association to thrombotic events, however, statistical significance for this association was not found in the multivariant analysis [46]. In contrast, there are several studies that despite having shown the high prevalence of aPL and thrombosis in acute COVID-19 infection, they did not find an association between the two processes [43,53,54,55]. In the same way, no associations were found with skin manifestations suggestive of APS that are common in COVID-19, such as livedo reticularis and digital ischemia [56]. 

Ferrari et al. found a similar prevalence for LA, aB2GP1 and aCL in severe and non-severe COVID-19 patients [49,57,58]. Amezcua-Guerra et al. studied all criteria and extra-criteria aPL in 21 ICU patients, the IgM isotype being the most frequent one studied. However, they did not find any association between aPL presence and thrombotic events [44]. 

Some authors suggest that domain I of aB2GP1 is the main immunogenic epitope targeted by aB2GP1 antibodies in APS patients because it is strongly associated with thrombosis [63]. Thus, the fact that only 5% of COVID-19 patients with aPL recognize B2GP1 domain I suggests that aPL could be different from those detected in APS patients in the context of COVID-19 [27]. No clinical association of aPL in a CAPS-like situation was found. Only 8% prevalence of aPL (criteria and non-criteria) was found in a study of 35 patients who died from COVID-19 with signs of coagulopathy and multiorgan thrombosis in more than three organs at autopsy [59].

A multicenter study that analyzed aPL in COVID-19 patients showed that the prevalence and titers of aPL or LA were not consistently increased nor associated with thrombosis when measured at a single timepoint [45]. This high prevalence of aPLs in COVID-19 patients and their lack of association with the clinical manifestations of APS have suggested that these aPL could be an epiphenomenon. In the case of LA, its presence may be due to the anticoagulation administered to almost all patients with severe COVID-19 infection [60] or who are becoming positive in the context of acute infection [64]. 

Another important fact is that most studies were carried out on elderly patients and this population group has shown a higher prevalence of aPL [65] and other autoantibodies such as antinuclear antibodies [66]. Studies in general use blood donor controls that include the population between 18–65 years [67]. When COVID-19 patients have been compared with a control population of a similar age, no significant differences have been found in the prevalence of aPL [52]. 

Criteria aPL, especially aCL, have been reported in the context of infectious diseases [68]. In addition, aPL in COVID-19 very rarely recognize domain I of B2GP1 [27]. The clinical association of IgM isotype aPL with thrombosis is quite controversial. Some authors have not found any clinical association with this isotype [69]. However, it is possible that the presence of IgG and IgA isotypes at the beginning of the symptoms can be explained by the fact that they were already preformed prior to infection. The class change from IgM to IgG or IgA is a process that requires a latency time, so it is unlikely that these antibodies are generated during infection. Moreover, the prevalence and titer of aPL do not change significantly when the acute phase of infection and post-convalescence state are compared. However, antibodies against SARS-CoV-2 antigens do increase dramatically. These facts suggest that the presence of aPL is independent of infection in most patients with aPL [52]. 

There has been controversy regarding the validity of the results for extra-criteria aPL, for example IgA aB2GP1. Due to the lack of standardization of the different tests available, there is a wide range of heterogeneity because the results depend on the system chosen to detect these antibodies [70]. At present, reliability can only be obtained with the use of properly accredited solid phase assays (ELISA). A large proportion of false negative tests was obtained when semi-solid phase systems (based on antigens-coated beads) were used [27,71]. However, the heterogeneity of the results in the case of aPS/PT was less because most of studies used the same ELISA kit (the first kit which was available). It should be noted that most of the studies on aPL in COVID-19 have been performed with small patient samples.

## 6. aPL Persistence

Although a second measurement at least 12 weeks apart has been recommended in the classification criteria, very few of the aPL studies in COVID-19 evaluated the two aPL samples with this separation. Contrary to what was found regarding the LA levels, the levels of aCL and aB2GP1 antibodies do not present significant variations in a second measurement [53,61].

However, when Sciascia et al. compared the aPL profile (criteria aPL and aPS/PT IgG and IgM) of 87 COVID-19 patients with APS patients and with patients with acute infections (excluding SARS-CoV2), they found that the aPL profile in COVID-19 patients differed from that of APS patients but was similar to those suffering from other infections [62]. In their first measurement, they found that although 52.9% of COVID-19 patients were positive for at least one aPL (29% LA positive, 10.3% positive for 2 or more aPL), no thrombotic events were observed in these patients. When they retested 12 patients from the COVID-19 group by solid phase assay (more than 12 weeks apart) they found that these patients were negative on the second measurement.

In a cohort of 361 COVID-19 patients evaluated for aPL in the first days of infection and reevaluated in a second sample after more than 12 weeks, Gil-Etayo et al. found no significant differences in the aPL prevalence between the first and the second samples and found a strong agreement between both determinations for criteria aPL (Weighted kappa: 0.85) and for IgA aB2GP1 antibodies (Weighted kappa: 0.91). However, concordance in measurements of anti-PS/PT antibodies was weak (Weighted kappa 0.43–0.52) [52].The low agreement between aPS/PT samples could be explained by the already-described correlation between LA and aPS/PT antibodies [32].

## 7. Role of B2GP1 Levels

B2GP1, also known as Apolipoprotein H (ApoH), is a pleiotropic protein involved in coagulation (its anticoagulant function predominating) [72] B2GP1 is also involved in inflammation, complement regulation and in the elimination of the circulation of microorganisms, necrotic cells and apoptotic bodies [72,73,74]. B2GP1 is involved in various biological pathways of innate and adaptive immunity through its effect on complement and coagulation cascades and its scavenger capacity [73,75,76,77]. This scavenger function plays an important role in the elimination of the excess of apoptotic bodies, the accumulation of which has been found to increase prothrombotic activity [14]. This function could imply an indirect anticoagulant activity.

The B2GP1 protein that is composed of five domains of the family of complement regulatory proteins is mainly synthesized in the liver [78]. Physiologically, B2GP1 is located principally in serum and the placenta and binds to activated or damaged endothelium (it is absent in intact endothelium). The placenta is the tissue with the highest concentration of B2GP1 [79]. In B2GP1 knockout mice, a reduction in embryo implantation has been observed as well as lower fetal/placental weight, which suggests defective placentation in these animals [80]. Likewise, mutation in the ApoH gene that impairs B2GP1 production in patients is associated with recurrent thrombosis [81]. Furthermore, early-onset preeclampsia and variations in placental oxygenation are more frequent in women with B2GP1 deficiency [82,83,84]. This reinforces the functional value of B2GP1 as a scavenger and a coagulation regulatory protein. More studies are mandatory to better understand its participation in physiological and pathological processes to prevent it from only being considered as an autoantigen for antiphospholipid antibodies in the antiphospholipid syndrome [72,78,85]. 

The coexistence of antibodies and antigen in the blood of aPL carriers allows for the formation of circulating immune complexes (CIC) composed of aPL bounded to B2GP1. The presence of CIC has been strongly associated with the risk of thrombotic events and with APS extra-criteria manifestations [86,87]. Approximately one fourth of the patients who are aPL positive are also CIC positive. It has been observed that CIC levels become elevated prior to the thrombotic event and decrease after it [88]. Interestingly, a total absence of CIC has been observed in patients with COVID-19 who are aPL positive [46]. This absence could be due to the fact that COVID-19 patients have very low serum levels of B2GP1 and that these low levels could lead to an imbalance in the concentrations of antigen and antibodies that would prevent the formation of CIC. The presence of very low levels of B2GP1 has also been described in other acute infections, in sepsis and in disseminated intravascular coagulation. Hence, these low levels behave as a negative acute phase reactant [85]. In COVID-19 patients the decrease in blood B2GP1 levels has been associated with a higher risk of ventilatory failure [46] as well as a greater predisposition for sepsis and mortality in ICU patients [89]. This suggests that both a decreased production or high consumption of the protein could occur in situations of organic stress, resulting in a lack of CIC detection due to the fact that the necessary antigens are not available for binding to the antibodies [90]. This hypothesis has been supported by the results of some studies carried out. 

The expression of the genes involved in angiogenesis was evaluated in a study carried out in patients who died due to COVID and a decrease in the apoH gene was found. In addition, thrombosis was found on a regular basis in the autopsies of these patients [91]. The presence of low blood levels of B2GP1 in the early stages of COVID-19 has been described using various methodologies such as mass spectrometry and EIA [46,92]. The study by Geyer et al. prospectively assessed the levels and found that they recover during convalescence [92]. Low levels of B2GPI are associated with the occurrence of thrombosis, although the mechanisms involved are unknown. Zhang et al. reported that low B2GP1 levels seen in patients with partial B2GPI deficiency (missense mutation) are associated with recurrent thrombosis [93]. This work confirmed previous observations that had reported that patients with the B2GPI H3 haplotype (with lower levels of plasmatic protein) have a greater capacity to generate thrombin. Carriers of this haplotype H3 have seven times more venous thrombosis than carriers of the B2GPI H1 haplotype (present in 85–90% of the general population) [81]. Patients in the early stages of COVID-19 would react in a way similar to an acquired partial deficiency of B2GPI triggered by the infection. During recovery, this deficiency corrects itself, the patients recovering their B2GP1 levels in blood.

Paradoxically, the risk of thrombosis in patients with total B2GP1 deficiency is not greater than the risk in the general population. Thrombophilia is also not observed in knockout mice for apoH gene [94,95]. However, it has been observed that the number of embryos that reach term in the pregnancies of these mice is significantly lower than in wild-type mice [80]. Both facts could be explained by the genetic robustness: other genes are used that give rise to alternative molecules that supply the functions of the original protein, avoiding the deleterious effects of its absence [96]. Only the embryos that use gene conversion survive [80]. 

In the first stages of COVID-19, the lower availability of serum B2GP1 would result in a lower amount of the protein adhered to the membranes of platelets and endothelium. This would reduce the capacity for indirect activation of receptors mediated by aPL, one of the main pathogenic mechanisms of aPL [97]. 

Likewise, a synergistic effect could be seen due to B2GP1 deficiency in the case of patients with COVID-19 in the presence of aPL [51]. B2GP1 deficiency could be a possible new mechanism of APS-related thrombosis. However, more investigations should be performed to better understand the underlying pathogenic mechanisms.

## 8. Management of aPL Carriers with COVID-19 Infection

At the time this current article is being written, it is difficult to establish evidence-based recommendations for patients with COVID-19 who are carriers of aPL. The levels of evidence are low because most of the studies carried out are retrospective and include small populations. According to the work published by Gil-Etayo et al. that includes the largest sample studied up to date with a second systematic aPL determination after 12 weeks to date, the aPL levels with the exception of LA would hardly change. Therefore, a single positive measurement of any aPL except that of lupus anticoagulant would be sufficient to classify an aPL carrier. Hence, it may not be necessary to wait 12 weeks to perform a second confirmation measurement. With the exception of LA that generally became negative in a second measurement, these measurements remain constant.

A series of recommendations has been proposed by the APS ACTION group for the management of aPL-positive patients within the context of COVID-19 disease in accordance with whether they are criteria or extra-criteria aPL [98]:

1. If there is no history of thrombosis, the group recommends the starting of anticoagulation with LMWH at prophylactic doses when there is an association with other additional prothrombotic factors or if the patient requires hospital admission.
2. When there is a history of thrombosis or in patients with anticoagulant treatment at a prophylactic dose, they recommend adapting the LMWH to therapeutic doses or that oral anticoagulants be maintained with close monitoring of INR.
3. Anticoagulation with LMWH at a prophylactic dose should be considered until the end of the puerperium in pregnant patients without other risk factors. The established dose should be continued if these patients have a history of previous anticoagulation. However, in those patients who have not had a prior indication for anticoagulation, this treatment should be discontinued once they are overcome the COVID-19 disease.

## 9. Discussion

COVID-19 has been associated with a high prevalence of both criteria and extra-criteria aPL. However, the million dollar question is whether they really have any clinical relevance in both patients with transient positivity and in those with permanent positivity. The most accepted hypothesis seems to be that it would be an epiphenomenon secondary to the infection. However, there is no unanimity in considering it this way because many of the studies carried out have biases that may be masking the real role of aPL in COVID-19. The prospective study by Gil-Etayo et al. has suggested that there may be two different mechanisms in patients associated with COVID-19 who are carriers of aPL regarding the appearance of thrombosis.

One mechanism may be the risk of thrombosis during the first days of infection inherent to the prothrombotic status in the SARS-CoV2 infection. Another one is that aPL carriers would have an additional (and later) mechanism of suffering a thrombotic event. The presence of aPL (first hit) in the context of the intense inflammatory activity that occurs in COVID-19 (second hit) would trigger a thrombotic event. Thus, aPL would have an additive effect on the risk of thrombosis generated by the infection itself.

In COVID-19 infection, high prevalence of classic aPL has been described. However, when expanded aPL screening has been performed, extra-criteria aPL are even more prevalent in numerous studies [27,41,46,62]. Therefore, it would be very important to determine both the criteria aPL and non-criteria aPL in COVID-19 patients as early as possible since they have a higher risk of thrombosis than patients without aPL and to identify this population group for early therapeutic intervention. To confirm these hypothesis, rigorous studies including a large number of patients, complete screening for aPL that includes extra-criteria, systematic confirmation of aPL 12 weeks apart should be performed. In addition, aPL must be determined using standardized solid phase techniques, which is very important to detect extra-criterion aPL and to avoid false negatives.

Looking ahead to the future, it should be kept in mind that as the incidence of COVID-19 worldwide is currently decreasing, it would become more difficult to carry out new prospective studies on a methodological level that would provide more evidence than that which has already been published.

However, the systematic collection of patients and their samples that has been carried out in most hospital centers could lead to future multicenter studies with large COVID-19 cohorts. This could provide better understanding regarding both the behavior of antiphospholipid antibodies and PSA in the context of acute SARS-CoV2 infection.

The limitations of this review: 

Section 4. Various biases have occurred in most of the available publications due to the methodological design. The study has been mainly limited to hospitalized COVID-19 patients, this resulting in a selection bias since they represent a small percentage of the total COVID-19 population. Biases have also been committed when the aPL was determined as not all the criterion and extra-criterion aPL were systematically performed on all the patients. Furthermore, the diagnostic kits were not used homogeneously so that many prevalence amounts may be both over- or underestimated.

In Section 5, the population samples studied are too small in most of the publications, so that statistical hypothesis testing errors, both type I error (rejection of a true null hypothesis) and type II error (the mistaken acceptance of a false null hypothesis) may exist

In Section 6, although the classification criteria recommend a second determination of aPL 12 weeks later, this was only done in a few studies and only in a selection of patients. There was only one study that had done it systematically [52].

In regard to Section 7, the physiological functions of B2GP1 are currently not well known. Their involvement in coagulation is controversial, since both procoagulant and anticoagulant functions have been described. At present, it is not possible to confirm that a deficit of B2GP1 in the blood implies a prothrombotic state. In addition, very few studies have been carried out on the B2GP1 levels in disease, so that new studies are needed to confirm these hypotheses.

The recommendations made in Section 8 and Section 9 are based on studies that mostly included small cohorts with weak statistical associations. However, the methodology used was the best possible available at the time of the study, given the collapse of the health system that occurred during the pandemic and the urgent need for new information to improve the knowledge of COVID-19 disease.

## 10. Conclusions

1. A high prevalence of antiphospholipid antibodies (aPL) has been observed in patients with acute COVID-19. This could be due to the high proportion of elderly patients in these studies. However, these differences are reduced when compared with control groups of similar ages since they are more predisposed to developing autoantibodies.
2. Antiphospholipid antibodies except for lupus anticoagulant persist over time in patients with SARS-CoV2 infection.
3. Most of the studies carried out on antiphospholipid antibodies in COVID-19 patients have been conducted in small population samples and have only determined the classic antibodies.
4. Extra-criteria aPL can be even more prevalent than classic aPL, so they should be included in the screening.
5. B2GP1 deficiency could be a criterion for increased susceptibility to complications in patients with severe infections such as severe sepsis and COVID-19.
6. Coagulopathy secondary to COVID-19 is an independent entity. However, it constitutes an additional prothrombotic factor in patients with preformed aPL. APL-related thromboses occur later than those related to SARS-CoV2 infection.

## Figures and Tables

**Table 1:** Set of studies on aPL presence in COVID-19 patients. The prevalence and types of aPL, confirmation after 12 weeks and the association with thrombosis were analyzed. *LA: lupus anticoagulant; R: Retrospective; P: Prospective; Evidence level: According to Miyakis* et al. [28].

Author	Type	No. of Patients	Confirmation > 12w	Criteria aPL	Extra Criteria aPL	Thrombosis Association	Evidence Level (6)
Zhang et al. [41]	Case report	3	N	Yes	Yes	Yes	IV
Helms et al. [42]	P	150	N	Yes (only LA)	N	Yes	I
Bowles et al. [43]	P	35	N	Yes (only LA)	N	N	II
Amezcua-Guerra et al. [44]	R	21	N	Yes	Yes	N	II/III
Borghi et al. [27]	P	122	N	Yes	Yes	N	I
Gatto et al. [45]	R	122	N	Yes	Yes	N	II/III
Serrano et al. [46]	P	474	N	Yes	Yes	Yes	I
Xiao et al. [47]	R	66	N	Yes	Yes	Yes	III
Hasan Ali et al. [48]	R	64	N	Yes	Yes	N	III
Frapard et al. [49]	R	68	N	Yes	Yes	Yes	III
Gazzaruso et al. [50]	R	45	N	Yes	N	Yes	III
Zuo et al. [51]	R	172	N	Yes	Yes	Yes	I
Gil-Etayo et al. [52]	P	362	Yes	Yes	Yes	Yes	I
Siguret et al. [53]	P	74	N	Yes	N	N	II
Devreese et al. [54]	P	31	N	Yes	Yes	N	II
Atalar et al. [55]	R	73	N	Yes	N	N	II
Gasparini et al. [56]	R	173	N	Yes	Yes	N	I
Ferrari et al. [57]	P	89	N	Yes	N	N	II
Trahtemberg et al. [58]	R	22	N	Yes	Yes	N	III
Previtali et al. [59]	R	35	N	Yes	Yes	N	III
Galeano-Valle et al. [60]	P	24	N	Yes	N	N	III
Vollmer et al. [61]	P	79	Only some patients	Yes	Yes	Yes	II
Sciascia et al. [62]	P	87	Only some patients	Yes	Yes	N	II

## Data Availability

Not applicable.
